# *Bacillus amyloliquefaciens GB03* augmented tall fescue growth by regulating phytohormone and nutrient homeostasis under nitrogen deficiency

**DOI:** 10.3389/fpls.2022.979883

**Published:** 2022-10-06

**Authors:** Qian Wang, Er-Ling Ou, Pu-Chang Wang, Ying Chen, Zi-Yuan Wang, Zhi-Wei Wang, Xiang-Wen Fang, Jin-Lin Zhang

**Affiliations:** ^1^ State Key Laboratory of Herbage Improvement and Grassland Agro-ecosystems, College of Ecology, Lanzhou University, Lanzhou, China; ^2^ Guizhou Institute of Prataculture, Guizhou Academy of Agricultural Sciences, Guiyang, China; ^3^ School of Life Sciences, Guizhou Normal University, Guiyang, China; ^4^ State Key Laboratory of Herbage Improvement and Grassland Agro-ecosystems, College of Pastoral Agriculture Science and Technology, Lanzhou University, Lanzhou, China

**Keywords:** *Bacillus amyloliquefaciens* GB03, plant growth promotion, nitrogen deficiency, phytohormone, nutrient, tall fescue

## Abstract

Nitrogen is an important nutrient for plant growth and development. Soil microorganisms have been used to curb the imbalance between the limited content of natural environmental nitrogen and the pollution caused by increasing nitrogen fertilizer use in ecologically fragile areas. *Bacillus amyloliquefaciens* GB03 has been shown to confer growth promotion and abiotic stress tolerance in *Arabidopsis thaliana*. This study provided a new insight into the role of the plant growth-promoting rhizobacterium *B. amyloliquefaciens* GB03 as an initiator of defense against nitrogen deficiency in non-leguminous grass tall fescue (*Festuca arundinacea*). Two-week-old seedlings of tall fescue were grown with or without GB03 for 4 weeks under total nitrogen (3.75 mM NO_3_^-^) or low nitrogen (0.25 mM NO_3_^-^) treatment. Growth parameters, chlorophyll content, endogenous total nitrogen, total phosphorus content, and phytohormone content, including those of auxin indole-3-acetic acid, cytokinin, gibberellic acid, and abscisic acid, were determined at the time of harvest. Tall fescue grown in GB03-inoculated soil was more robust than the non-inoculated controls with respect to plant height, root length, plant biomass, chlorophyll concentration, and nutrient (total nitrogen and total phosphorus) contents under total nitrogen treatment. GB03 increased indole acetic acid content by 24.7%, whereas decreased cytokinin and abscisic acid contents by 28.4% and 26.9%, respectively, under a total nitrogen level. Remarkably, GB03 increased indole acetic acid content by more than 80% and inhibited abscisic acid production by nearly 70% under a low nitrogen level. These results showed, for the first time, that GB03 played a crucial role in mediating NO_3_^–^dependent regulation of tall fescue growth and development, especially revealing the mechanism of soil bacteria improve resistance to nitrogen deficiency stress in non-nitrogen-fixing species.

## Introduction

In southwest China, cold-season turf grasses have received more attention owing to their longer green period. Tall fescue (*Festuca arundinacea*) belongs to the family Poaceae, subfamily Pooideae, tribe Poeae, and is a major cool-season turf grass and forage species worldwide. Additionally, because of its rapid growth, wide adaptability, strong regeneration, and long green period, it plays an important role in water and soil conservation, environmental protection, and ecological restoration (Yang et al., 2013; Tang et al., 2015). In previous studies, we collected several tall fescues from various lacations for cross-breeding. After years of comprehensive evaluation of drought, heat resistance, and production performance, we finally selected a new national forage grass variety “Qiancao No. 1” (registration number: 299) with superior indicators. Nevertheless, consistent with other species of tall fescue, the cultivation and seed production of “Qiancao No. 1” strongly depended on nitrogen fertilizers.

Nitrogen (N), an important nutrient for plant growth and development, is both the main structural material constituting plant organisms (Amtmann and Armengaud, 2009) and a key catalyst in various plant physiological and metabolic processes (Orsel et al., 2006). Nitrogen is absorbed from the soil either in inorganic forms, such as nitrate or ammonium, or in organic forms, mostly as free amino acids (Vance, 2001; Miller and Cramer, 2004). However, nitrate is the major form absorbed, and its availability can fluctuate markedly, both spatially and temporally, especially in ecologically fragile areas (Liu and Wang, 2012; Zhao and Ma, 2014). Application of a nitrogen fertilizer in the soil can effectively increase the yield of plant and crop, while increasing nitrogen fertilizer use severely causes environmental pollution (Foyer and Ferrario, 1994; Nosengo, 2003), particularly in aquatic ecosystems (Pretty, 2008; Robertson and Vitousek, 2009) and the atmosphere (Davidson, 2009; Smith et al., 2012). Therefore, it is extremely important to find a new method for solving the contradiction between the limited effectiveness of natural environmental nitrogen and environmental pollution due to increased fertilizer use.

In terrestrial ecosystems, plants are producers and soil microorganisms are decomposers. Producers release photosynthetic products into the soil in the form of root secretions and plant residues, supplying soil microbial energy and carbon sources. On the other hand, soil microorganisms convert organic nutrients into inorganic nutrients, contributing to plant absorption and utilization (Marschnera and Timonen, 2005). Plant rhizosphere soil contains large numbers of microorganisms that play important roles in organic matter decomposition, nutrient recycling, and plant nutrient utilization. Some plant growth-promoting rhizobacteria (PGPR) can promote plant growth, prevent disease, and increase crop yield (Schippers et al., 1987; Kloepper, 1992). Therefore, these bacteria have been widely applied in agriculture to increase seed emergence, improve plant weight and crop yield, promote efficient uptake and utilization of nutrients, and enhance disease resistance (Zhang et al., 2008a; Zhang et al., 2008b; Paré et al., 2011).

*Bacillus subtilis* GB03, now renamed as *Bacillus amyloliquefaciens* GB03, is a commercially available PGPR strain that can be introduced into the soil at the time of planting *via* seed coating (Choi et al., 2014). Since the mid-1990s, researchers had focused on the biotic stress control by GB03 firstly. Raupach and Kloepper (1998) found that GB03 enhanced the biological control of multiple cucumber pathogens. In *Arabidopsis thaliana*, many researchers provide new insight into the role of GB03 as an elicitor of defense responses (Pieterse et al., 2002; Ryu et al., 2004, Ryu et al., 2005; Rudrappa et al., 2010). In tomato (*Solanum lycopersicum*), the results of the present study suggested that integrated control of Fusarium crown and root rot could be achieved by combining the use of PGPR strains GB03 with plant disease inducers or conventional fungicides (Myresiotis et al., 2011). In the following, many studies had been conducted on composition analysis of the release, mechanism of action, and signal transduction pathways of GB03. Ryu et al. (2003) pointed out that GB03 could release volatile organic compounds (VOCs), devoid of classic phytohormones, which were capable of promoting plant growth. These VOCs also activated the differential expression of approximately 600 transcripts related to cell wall modifications, primary and secondary metabolism, stress responses, and hormone regulation (Zhang et al., 2007). Among these VOCs, methyl jasmonate, methyl salicylate, 2,3-butanediol, and acetoin had been found to trigger induced systemic resistance (ISR) and protect plants against pathogenesis to increase the plant growth (Ryu et al., 2004, Ryu et al., 2005). Studies had also shown the potential of GB03 to control iron acquisition and light energy conversion and emphasize the sophisticated integration of microbial signaling in photosynthetic regulation (Zhang et al., 2008a; Zhang et al., 2009). Similar results had also been reported in cassava (*Manihot esculenta*), an important agricultural crop, in which iron accumulation was increased, and growth promotion and photosynthetic efficiency improvement were observed in greenhouse-grown plants exposed to GB03 (Freitas et al., 2015). In addition, GB03 augmented plant tolerance to salt and osmotic stresses by regulating tissue-specific expression of Na^+^ transporter to decrease Na^+^ uptake and accumulation and increasing osmoprotectant accumulation in many species (Zhang et al., 2008b; Zhang et al., 2010; Han et al., 2014a; Han et al., 2014b; Niu et al., 2016; Han et al., 2017).

Recently, it has been observed that bacterial volatile components can serve as agents for triggering growth promotion through many pathways. However, the ability of PGPR to induce nitrogen assimilation *via* established operational mechanisms in non-leguminous species, particularly the role of PGPR *B. amyloliquefaciens* strain GB03 in regulating gramineous plant growth and development, has not been reported. In this study, we reported a novel mechanism in which the bacterial strain GB03 augmented tall fescue growth by increasing the chlorophyll and nutrient contents and regulating the nitrogen-dependent homeostasis of phytohormones. We also proposed a new paradigm for PGPR to mediate nitrogen deficiency adaptation in non-nitrogen-fixing gramineous plants.

## Materials and methods

### Bacterial culture

PGPR strain *Bacillus amyloliquefaciens* GB03 was streaked onto Luria broth (LB) agar plates and incubated at 28°C for 24 h without light. PGPR cells were harvested from LB agar plates using double-distilled water (DDW) to yield 10^9^ colony-forming units (CFU) mL^-1^, as determined by optical density and serial dilutions with plate counts (Zhang et al., 2007; Zhang et al., 2008b).

### Plant materials and treatments

Seeds of tall fescue (*Festuca arundinacea*) were obtained from our breeding line ‘Qiancao No. 1’ and were surface sterilized with 5% (v/v) bleacher followed by 70% ethanol and washed five times with sterile water, then germinated at 25°C on moist filter paper for 2 days. Uniform seedlings were transplanted to plastic pots (diameter 10 cm, five seedlings per pot) containing autoclave-sterilized vermiculite. Six pots in one pallet (the pallet length 50 cm, width 25 cm, and depth 5 cm, we arranged eight pallets totally) were filled with Hoagland nutrient solution containing 1.25 mM KNO_3_, 0.25 mM KH_2_PO_4_, 0.5 mM MgSO_4_·7H_2_O, 1.25 mM Ca(NO_3_)_2_·4H_2_O, 11.6 µM H_3_BO_3_, 4.6 µM MnCl_2_·4H_2_O, 0.19 µM ZnSO_4_·7H_2_O, 0.08 µM CuSO_4_·5H_2_O, 0.12 µM Na_2_MoO_4_·2H_2_O, and 10 µM Fe(III)-EDTA. Nutrient solutions were renewed every 3 days. Seedlings were grown in the greenhouse under a daily photoperiod of 16/8 h (light/dark) with a light intensity of 230–300 μmol m^–2^·s^–1^, temperature of 25°C ± 2°C/23°C ± 2°C (day/night), and relative humidity of 60 ± 5%.

Two-week-old seedlings were treated under the following conditions: (i) total nitrogen (TN-3.75 mM NO_3_^-^): seedlings were grown in Hoagland nutrient solution and (ii) low nitrogen (LN-0.25 mM NO_3_^-^): seedlings were subjected to 0.25 mM NO_3_^-^ (Hoagland nutrient solution was deprived of KNO_3_ and Ca(NO_3_)_2_·4H_2_O; 1.25 mM KNO_3_ was substituted with 0.25 mM KNO_3_ and 1 mM KCl, while 1.25 mM Ca(NO_3_)_2_·4H_2_O was substituted with 1.25 mM CaCl_2_). After 4 days, the seedlings were inoculated directly into the vermiculite with 1 ml bacterial suspension or 1 ml DDW as a control, for another 4 weeks (the bacterial suspension or sterile water was supplemented every week).

### Plant biomass and physiological index measurements

Seven*-*week-old plants were removed from their pots, and their roots were rinsed with water to remove attached vermiculite. Plant height and root length were measured using a ruler. Thirty independent plants were measured for each treatment. Shoots and roots were separated and blotted, fresh weights (FW) were acquired immediately, and samples were dried in an oven at 70°C for 3 days to determine dry weights (DW). Eight independent biological replicates were used for each treatment.

The chlorophyll content was estimated according to the method of Porra et al. (1989). Fresh leaf samples were crushed thoroughly with 80% acetone in the dark and centrifuged at 9,000 g for 10 min at 4°C. The supernatant was collected, and absorbance was measured at 645 and 663 nm using a UV spectrophotometer (UV-2700, Unico Inc., Shanghai, China). Chlorophyll a, chlorophyll b, and total chlorophyll contents were estimated using the equations of Porra et al. (1989). Eight independent biological replicates were used for each treatment.

### Determination of total nitrogen and total phosphorus

Plant total nitrogen and total phosphorus contents were analyzed in the dried samples. Total nitrogen content was determined using semimicro-Kjeldahl digestion and distillation (Nelson and Sommers, 1980). Total phosphorus content was determined using the vanadomolybdate yellow method (Jackson, 1959). Eight independent biological replicates were used for each treatment.

### Indole-3-acetic acid, cytokinin, gibberellic acid, and abscisic acid measurements

Auxin indole-3-acetic acid (IAA), cytokinin (CK), gibberellic acid (GA), and abscisic acid (ABA) were determined using enzyme-linked immunosorbent assay (ELISA). In brief, according to the instructions of IAA, CK, GA, and ABA ELISA kits (Mibio, Shanghai, China), we extracted the phytohormone and measured the absorbance (OD value) at 450 nm using a microplate reader (BioTek Inc., USA). Each sample was assayed in three independent biological replicates, and each biological replicate was assayed in three technical replicates. Finally, the IAA, CK, GA, and ABA contents were calculated according to the standard liquid concentration.

### Statistical analysis

Statistical analyses were performed using 16.0 (SPSS Inc., Chicago, IL, USA). Duncan’s multiple-range test was used to detect significant differences between the means at a significance level of *p* < 0.05.

## Results

### GB03 promoted tall fescue growth under various nitrogen levels

**Figure 1:**
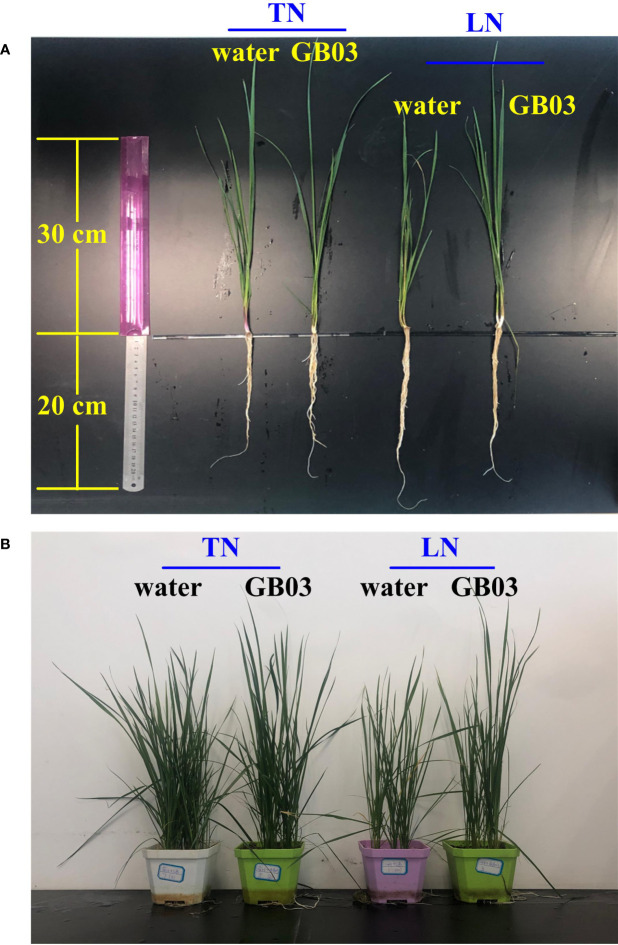
Effects of GB03 on tall fescue phenotype. **(A)** Individual plant and **(B)** one pot of plant (containing five seedlings) were photographed from different parallel treatments, including the total nitrogen level (TN-3.75 mM NO_3_^-^) and the low nitrogen level (LN-0.25 mM NO_3_^-^) with GB03, respectively, and the water treatment as control.

**Figure 2:**
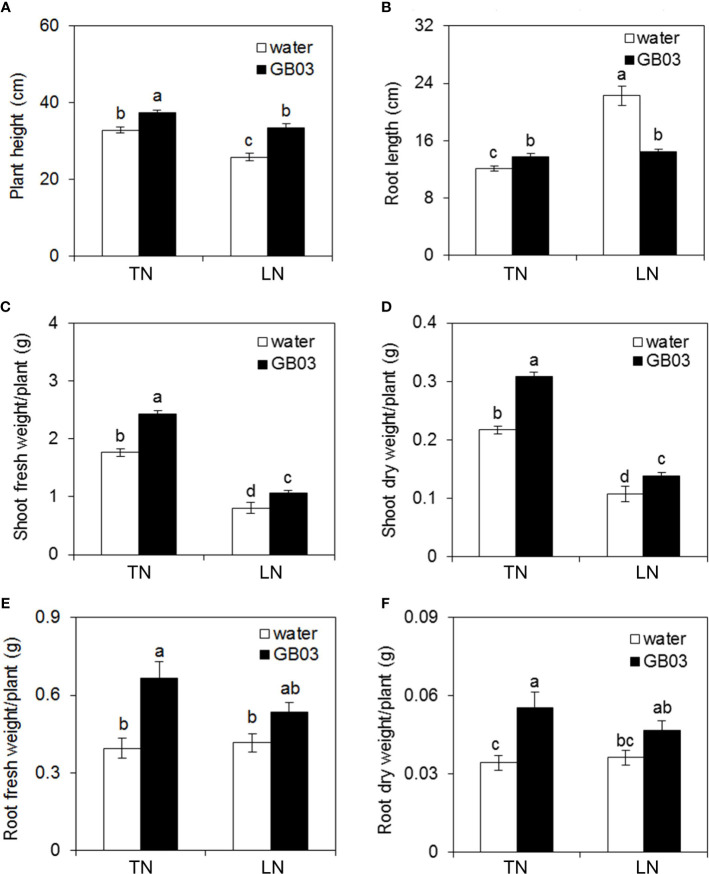
Effects of GB03 on tall fescue growth under various concentrations of NO_3_^-^. **(A)** Plant height, **(B)** root length, **(C)** shoot fresh weight, **(D)** shoot dry weight, **(E)** root fresh weight, and **(F)** root dry weight. Values are means ± SE (A and B *n* = 30; C-F *n* = 8), and bars indicate SE. Columns with different letters indicate significant differences at *p* < 0.05 (Duncan’s test).

The GB03-exposed plants were greener than water control, and this phenotype was more pronounced under LN conditions, because the part of foliage showed yellow under LN without GB03 (**Figure 1**). Compared with TN treatment, LN stress reduced plant height by 21.3% but promoted root length dramatically by 83.8% without GB03 (**Figures 2A, B**). The PGPR strain GB03 enhanced plant height by 14% and 29.7% under TN and LN treatments, respectively, compared with the non-inoculated controls (**Figure 2A**). Root length increased by 13.2% under TN treatment, whereas it decreased by 34.8% under LN stress with the addition of GB03 (**Figure 2B**). Compared with TN, LN reduced shoot FW and DW by more than 50% (**Figures 2C, D**) but had no effect on root biomass without GB03 (**Figures 2E, F**). GB03 increased biomass in both the shoots (FW by 37.6%, DW by 41.8%) and roots (FW by 68.5%, DW by 62%) under TN treatment; meanwhile, the biomass of shoots (FW by 32.9%, DW by 29%) and roots (FW by 28.4%, DW by 29.2%) was improved under LN stress, although the latter was not statistically significant (**Figures 2C–F**).

### GB03 enhanced nutrient accumulation in tall fescue under various nitrogen levels

**Figure 3:**
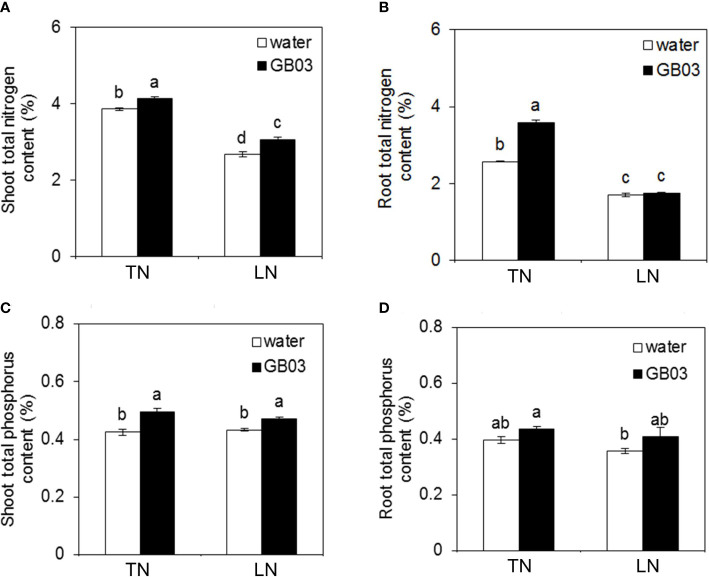
Effects of GB03 on N and P in tall fescue under various concentrations of NO_3_^-^. **(A)** Shoot total nitrogen content, **(B)** root total nitrogen content, **(C)** shoot total phosphorus content, and **(D)** root total phosphorus content. Values are means ± SE (*n* = 8), and bars indicate SE. Columns with different letters indicate significant differences at *p* < 0.05 (Duncan’s test).

Compared with the TN treatment, LN stress significantly decreased the shoot total nitrogen content by 30.8% without GB03; however, compared with water control, GB03 improved the shoot total nitrogen content by 7% under TN conditions, and the magnitude of increase under LN conditions (by 14.5%) was twice than under TN (**Figure 3A**). Consistent with that seen for the shoot, LN stress also reduced the root total nitrogen content by 39.4% compared with TN treatment without GB03; however, the root total nitrogen was enhanced by GB03 exposure under TN treatment (**Figure 3B**). On the other hand, phosphorus showed different changes from nitrogen under various treatments. First, the NO_3_^-^ supply from 3.75 to 0.25 mM had no effect on total phosphorus content in neither shoot nor root without GB03, whereas plants with GB03 exposure had improved total phosphorus content in shoot under TN (by 16.5%) and LN (8.7%) treatments (**Figure 3C**). Root total phosphorus content was maintained at a steady level under various treatments (**Figure 3D**).

### GB03 increased chlorophyll content in tall fescue under various nitrogen levels

Compared with the TN treatment, LN stress reduced the chlorophyll a content (by 18.5%) and the total chlorophyll content (by 15.5%) but had no effect on the chlorophyll b content without GB03 (**Table 1**). Nevertheless, regardless of TN treatment or LN stress, chlorophyll a, chlorophyll b, and total chlorophyll content increased by nearly 30% with GB03, compared with the water control (**Table 1**).

**Table 1:** The chlorophyll *a*, *b* and total chlorophyll concentrations in tall fescue.

Treatments	Chlorophyll *a*(mg g^-1^ FW)	Chlorophyll *b*(mg g^-1^ FW)	Total chlorophyll(mg g-^1^ FW)
TN	1.08 ± 0.04^b^	0.34 ± 0.01^bc^	1.42 ± 0.07^b^
TN + GB03	1.36 ± 0.02^a^	0.44 ± 0.02^a^	1.80 ± 0.02^a^
LN	0.88 ± 0.02^c^	0.33 ± 0.02^c^	1.20 ± 0.03^c^
LN + GB03	1.17 ± 0.06^ab^	0.40 ± 0.01^ab^	1.57 ± 0.06^ab^

The treatments were total nitrogen (TN), total nitrogen with soil bacteria GB03 (TN + GB03), low nitrogen stress (LN), and low nitrogen stress with soil bacteria GB03 (LN + GB03). Values are means ± SE (*n* = 8). The different letters indicate significant differences at *p* < 0.05 (Duncan’s test).

### GB03 regulated pyhormone contents in tall fescue under various nitrogen levels

**Figure 4:**
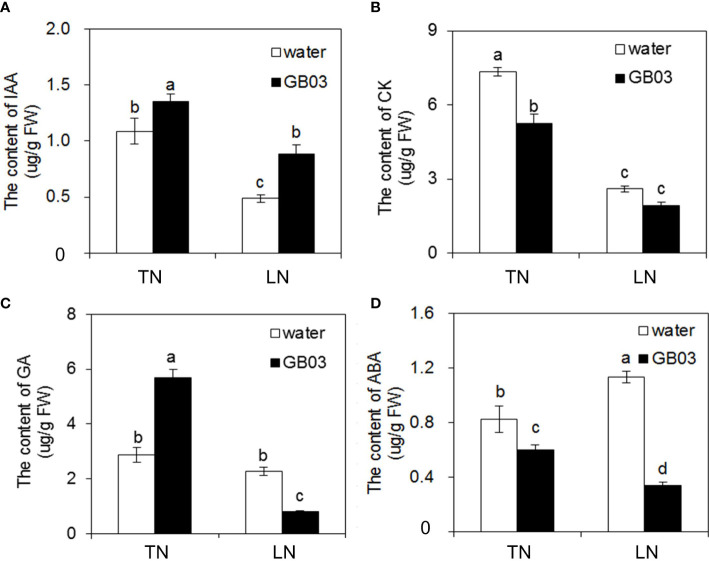
Effects of GB03 on phytohormone content in tall fescue under various concentrations of NO_3_^-^. **(A)** Indole-3-actetic acid (IAA) content, **(B)** cytokinin (CK) content, **(C)** gibberellic acid (GA) content, and **(D)** abscisic acid (ABA) content. Values are means ± SE (*n* = 9), and bars indicate SE. Columns with different letters indicate significant differences at *p* < 0.05 (Duncan’s test).

In non-GB03-exposured plants, LN stress reduced IAA and CK contents by 55.1% and 64.5%, respectively (**Figures 4A, B**), while it had no effect on GA content (**Figure 4C**) compared with TN treatment. On the contrary, ABA content was increased by 37.5% by LN stress versus TN treatment (**Figure 4D**). With GB03 exposure for 4 weeks, an increase in IAA content was observed under TN by 24.7% and under LN by 81.8% (**Figure 4A**). In contrast to IAA, the CK and ABA contents were reduced with GB03 exposure both under TN and LN treatments, and the decreasing degree of ABA content in the latter (nearly 70%) was larger than that in the former (no more than 30%) (**Figures 4B, D**). On the other hand, although NO_3_^-^ levels had no effect on GA content in tall fescue without GB03, GA content responses varied with different NO_3_^-^ concentrations under GB03 compared with the water control. GA increased by 98% under TN and decreased by 65% under LN (**Figure 4C**).

## Discussion

### *B. amyloliquefaciens* GB03 enhanced tall fescue growth under nitrogen deficiency stress

*B. amyloliquefaciens* GB03, as a PGPR strain, promoted plant growth in many plant species, including *Arabidopsis thaliana* (Ryu et al., 2003; Zhang et al., 2008a; Xie et al., 2009; Paré et al., 2011), wheat (*Triticum aestivum*) (Zhang et al., 2014), alfalfa (*Medicago sativa*) (Han et al., 2014a), white clover (*Trifolium repens*) (Han et al., 2014b), cassava (*Manihot esculenta*) (Freitas et al., 2015), *Puccinellia tenuiflora* (Niu et al., 2016), and *Codonopsis pilosula* (Han et al., 2017). The growth promotion of tall fescue by *B. amyloliquefaciens* GB03 inoculation in the soil evaluated in our study was consistent with the above reports (**Figure 1**). Here, in order to prove that the growth promotion of GB03 had a certain strain specificity, we chose *Escherichia coli* strain DH5α as positive control at the initial growth experiment, finding that DH5a failed to augment growth with respect to plant height, root length, shoot and root FW, and DW regardless of TN or LN treatment (**Figure S1**, **Table S1**). Contrary to DH5α, compared with water control, the plant height, root length, and shoot and root FW and DW increased to varying degrees with GB03 TN conditions (**Figure 2**). In addition, inducible plant growth promotion mediated by GB03 had also been observed under some conditions, including salinity stress (Zhang et al., 2008b; Zhang et al., 2014; Han et al., 2014a; Han et al., 2014b; Niu et al., 2016; Han et al., 2017), iron deficiency (Zhang et al., 2009; Freitas et al., 2015), and osmotic stress (Zhang et al., 2010), which could ultimately enhance plant stress resistance. However, the ability of GB03 to improve plant adaptability to nitrogen deficiency has not been previously reported. In the present study, the shoot FW and DW of tall fescue decreased by more than 50% from TN to LN without GB03 treatment (**Figures 2C, D**), and the shoot total nitrogen content also decreased by 30.8% (**Figure 3A**), indicating that nitrogen deficiency dramatically hindered shoot biomass accumulation in plants. When *B. amyloliquefaciens* GB03 was inoculated into the soil, the plant height and shoot FW and DW of tall fescue were increased under both TN and LN conditions; remarkably, the latter was twice as much as the former (**Figures 2A, C, D**). This was in line with the changes in shoot total nitrogen content, which was also elevated by GB03 and increased under LN, twice that of TN, compared with the water control (**Figure 3A**). The results presented here suggested that GB03 could regulate the distribution of nitrogen and improve the nitrogen deficiency resistance of grass by enhancing the accumulation of shoot total nitrogen. In addition, GB03 promoted shoot growth, possibly because it could enhance the accumulation of shoot total phosphorus, another essential macronutrient for plant growth and development (**Figure 3C**). A similar study pointed out that GB03 could improve *A. thaliana* salt tolerance by regulating the tissue-specific expression of genes to accumulate the less toxic Na^+^ (Zhang et al., 2008b). Although our study also found that the growth-promoting effect of GB03 under nitrogen deficiency was related to the improvement of nutrient accumulation at the physiological level, the transporters involved in this process require further in-depth research. Intriguingly, unlike GB03 which promoted root growth under TN conditions, it had no effect on root FW and DW under LN conditions (**Figures 2E, F**), and root total nitrogen and phosphorus content did not change between the water control and GB03 treatments (**Figures 3B, D**). In these contexts, the growth-promoting effect of GB03 was different for the two nitrogen levels: under TN conditions, GB03 promoted tall fescue growth not only by enhancing nutrient (nitrogen and phosphorus) accumulation in the shoot but also by facilitating nitrogen uptake in the root; under LN conditions, GB03 preferentially promoted nutrient (nitrogen and phosphorus) accumulation in the shoot, rather than root uptake, to resist nitrogen deficiency stress. This functional difference may be due to GB03-induced genes, encoding nutrient transporters, for which expression was NO_3_^–^dependent.

*B. amyloliquefaciens* GB03 also affects photosynthesis to varying degrees in plants. Photosynthesis converts light energy into chemical energy in the form of energy-rich sugar. The sugars produced serve not only as carbon and energy sources but also as pivotal signaling molecules for plant growth, development, and stress responses (Zhang et al., 2008a). In cassava, improved photosynthetic efficiency by increasing iron accumulation had been observed in greenhouse-grown plants exposed to GB03 (Freitas et al., 2015). In *Arabidopsis*, the potential of GB03 to control iron acquisition in plants and emphasize the sophisticated integration of microbial signaling in photosynthetic regulation were demonstrated (Zhang et al., 2009). GB03 enhanced the photosynthetic capacity of *Arabidopsis* by improving the efficiency of the conversion of light energy, as well as by enhancing the photosynthetic apparatus (including increase in photosynthetic efficiency and chlorophyll content) (Zhang et al., 2008a) Consistent with GB03 improving salt resistance by directly and indirectly regulating plant chlorophyll content (Han et al., 2014a; Han et al., 2014b; Han et al., 2017), in addition to improving the accumulation of nutrient, GB03 augmented tolerance to nitrogen deficiency stress by enhancing tall fescue photosynthetic capacity, as evidenced by increasing chlorophyll content (**Table 1**). This result at the physiological level was in accordance with a study at the molecular level, where the mining of microarray data identified several GB03-induced photosynthetic genes, such as chlorophyll a/b binding protein 165/180 (CAB2) and RuBisCO subunit binding proteins (Zhang et al., 2007).

### *B. amyloliquefaciens* GB03 regulated nitrogen-dependent phytohormone levels in tall fescue

**Figure 5:**
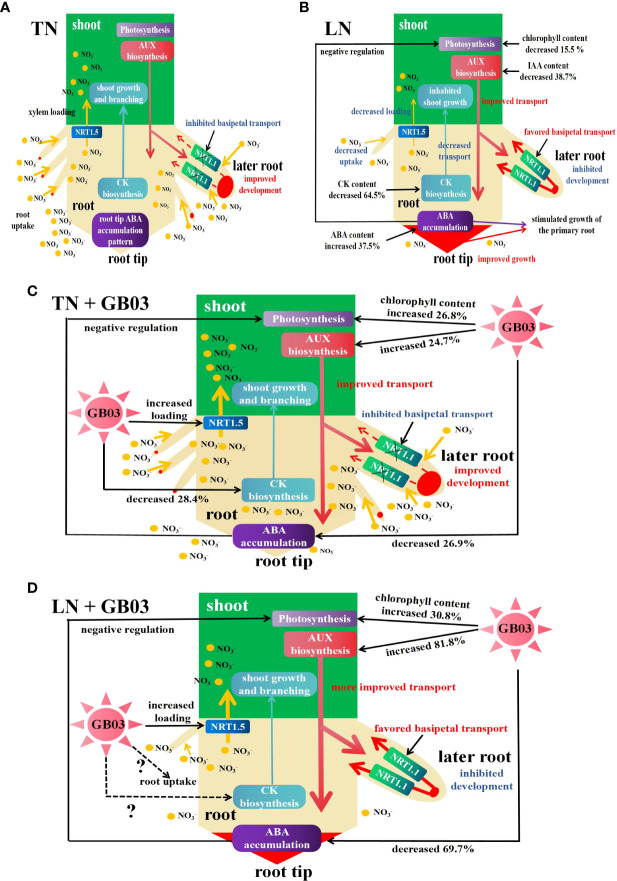
Schematic model for the interaction between nitrogen and phytohormones (auxin AUX, cytokinin CK, and abscisic acid ABA) in the regulation of tall fescue growth under different NO_3_^-^ treatments with or without GB03. **(A)** Under TN conditions, AUX was synthesized in the shoot and then was basipetally transported into the root, the NRT1.1 basipetal transport of AUX in lateral roots was inhibited, and AUX was accumulated in the later root tip, triggering the growth and development of later root and uptake of NO_3_^-^; CK was synthesized in the root and then was apically transported into the shoot, along with NO_3_^-^ loading by NRT1,5 from root to shoot, contributing to the shoot growth and branching; and NO_3_^-^ regulated the primary root growth *via* regulation of ABA accumulation in the root tip. **(B)** Under LN conditions, AUX translocated from shoot to root was stimulated by a decrease in NO_3_^-^ supply, along with NRT1.1 favoring basipetal transport of AUX in lateral roots, enhancing the accumulation of AUX in the primary root tip, which promoted the growth of the primary root versus inhibited the growth and development of the lateral root and impaired NO_3_^-^ uptake; the root biomass was decreased; CK biosynthesis and apical transport, as well as NO_3_^-^ loading by NRT1,5, were weakened by a decrease in NO_3_^-^ supply, and the shoot growth was inhibited; and root tip ABA accumulation was augmented to simulate growth of the primary root and impaired photosynthesis in shoot. **(C)** Under TN conditions, GB03 enhanced the AUX biosynthesis and basipetal transport in the shoot, and the NRT1.1 basipetal transport of AUX in lateral roots was inhibited, and AUX was accumulated in the later root tip, increasing the growth and development of later root and uptake of NO_3_^-^; CK content was decreased by GB03 which might contribute to stimulate NO_3_^-^ uptake by root, because of CK which had a negative regulation of nitrogen uptake-related genes; and the root tip ABA accumulation was decreased by GB03, which hindered the primary growth regulated by ABA. The abovementioned contributed to the increase in root biomass. Meanwhile, the reduction of ABA caused by GB03 negatively regulated photosynthesis, and GB03 enhanced chlorophyll content and NO_3_^-^ loading in the xylem, and the shoot growth was promoted. **(D)** Under LN conditions, the dual function of GB03 increased AUX biosynthesis and LN stimulated AUX basipetal transport in the shoot, leading to more AUX being accumulated in the root. However, the strong decrease in ABA accumulation caused by GB03 blocked the growth of primary root; this effect was greater than AUX accumulation which was induced by NRT1.1 basipetal transport in lateral roots. Finally, GB03 effectively alleviated primary root elongation at a low nitrogen level. Meanwhile, GB03 preferentially augmented shoot growth by improving photosynthesis and shoot total nitrogen content to resist low nitrogen stress.

Phytohormones were originally defined as a group of naturally occurring organic substances that influence plant growth and development at low concentrations (Kiba et al., 2011). In addition, phytohormones have been linked to various environmental responses to salt, drought, light, temperature, and nutrients (Zhu, 2002; Halliday et al., 2009; Patel and Franklin, 2009; Argueso et al., 2009). Auxin (IAA) is an important hormone that modulates numerous physiological processes in plants, contributing to their growth and development (Teale et al., 2006; Benjamins and Scheres, 2008; Vanneste and Friml, 2009). Auxin is mainly synthesized in the shoot and performs shoot-to-root phloem transport (Ljung et al., 2005). Modification in auxin content under various N treatments had been studied in many species, including wheat (*Triticum aestivum*) (Chen et al., 1998), soybean (*Glycine max*) (Caba et al., 2000), pineapple (*Ananas comosus*) (Tamaki and Mercier, 2007), maize (*Zea mays*) (Liu et al., 2010) and *A. thaliana* (Krouk et al., 2010). In each case, auxin seemed to be translocated from shoot to root was stimulated by a decrease in NO_3_^-^ supply. Therefore, it has long been a candidate for mediating nitrogen signals from shoot to root because of its basipetal transport and regulation of root growth and development (Forde, 2002; Jiang and Feldman, 2003; Fukaki and Tasaka, 2009). In *A. thaliana*, NRT1.1 favored basipetal transport of auxin in lateral roots, thus preventing auxin accumulation at the lateral root tip, which slowed the outgrowth and elongation of lateral roots in the absence of NO_3_^-^ (Krouk et al., 2010). In maize, there was a significant negative correlation between nitrate concentrations and IAA levels in the roots, and the primary root closest to the root tip (10 cm from the root tip) was the main zone in which the IAA level responded sensitively to nitrate supply, whereas primary root length showed a positive correlation with IAA content in roots (Tian et al., 2008). In these contexts, we proposed the mode for interaction between nitrogen and phytohormones in the regulation of tall fescue growth under the different NO_3_^-^ treatments (**Figures 5A, B**). LN firstly stimulated the transport of auxin from shoot to root, and NRT1.1 favored the basipetal transport of auxin in lateral roots, leading to auxin being accumulated in the primary root tip (**Figure 5B**), which promoted the growth of the primary root, while it inhibited the growth and development of the lateral root (**Figure 2B**). Inhibition of lateral root growth and development further hindered NO_3_^-^ uptake by roots, resulting in the decrease in root total nitrogen content (**Figure 3B**). NRT1.5 had been identified as an essential transporter in NO_3_^-^ long-distance transport from root to shoot in the xylem (Lin et al., 2008; Chen et al., 2012); its transport ability was weakened caused by its encoding gene *NRT1.5* being downregulated by LN in tall fescue root (these data were unpublished), decreasing the shoot total nitrogen content (**Figure 3A**). *B. amyloliquefaciens* GB03, as a PGPR, increased the IAA content under both TN and LN treatments (**Figure 4A**), indicating that GB03 induced IAA biosynthesis in tall fescue. GB03 induced IAA biosynthesis *via* a tryptophan-dependent pathway which was elucidated by transcript profiles. It was found that the transcripts of three nitrilases (which catalyze the terminal step in the tryptophan-dependent IAA pathway) were upregulated and transcripts of putative auxin efflux carriers were downregulated by GB03 exposure (Zhang et al., 2007). Additionally, auxin accumulation decreased in leaves and increased in roots with GB03 exposure, which was revealed in a transgenic DR5::GUS *Arabidopsis* line, suggesting that GB03 activated the basipetal transport of auxin in the shoot (Zhang et al., 2007). Here, based on the above, our model showed the role of GB03 in promoting tall fescue growth under different nitrogen levels (**Figures 5C, D**). Firstly, GB03 enhanced auxin biosynthesis in aerial regions and improved basipetal transport in the shoot; the amplification of IAA contents under LN conditions (increased by 81.8%) was much higher than under TN conditions (increased by 24.7%) (**Figures 5C, D**), which might be contributed to the GB03 dramatically induced tissue-specific gene expression of auxin-regulated genes under LN treatment. In addition, NRT1.5-mediated NO_3_^-^ long-distance transport was enhanced by GB03 regardless of TN or LN condition (these data were unpublished), increasing shoot total nitrogen content (**Figure 3A**) and shoot biomass (**Figures 2C, D**). However, it should be noted that GB03 had different effects on root growth under different NO_3_^-^ treatments. Under TN with GB03 treatment, the augments of root length and biomass were due to the increase in auxin biosynthesis and transport by GB03 and accumulation of auxin in both primary and later roots (**Figure 5C**). Under LN with GB03 treatment, despite the root biomass having had no change caused by auxin basipetal transport by NRT1.1 in later root (**Figure 5D**), GB03 effectively alleviated primary root elongation at a low nitrogen level (**Figure 2B**).

In addition, CK also controls various developmental processes in plants, such as cell division, senescence, gene expression, and nutritional signalings (Schmülling et al., 1997; Takei et al., 2002). CK is also well known for its ability to stimulate cell division and differentiation in the shoot (Kakimoto, 2003), and its level has frequently been found to correlate positively with the nitrogen status of the plant (Samuelson and Larsson, 1993; Wagner and Beck, 1993; Takei et al., 2001; Kiba et al., 2011). Moreover, it was well documented that nitrate induced cytokinin biosynthesis in roots, which was then transported from roots to shoots for systemic regulation (Sakakibara et al., 2006; Poitout et al., 2018). In our model, firstly LN hindered cytokinin biosynthesis in tall fescue root (**Figure 5B**), reducing the CK content (**Figure 4B**) and shoot growth (**Figures 2A, C, D**). Furthermore, CK also has a regulatory role in root architecture development, which has an opposing effect on shoots and roots. For example, CK stimulated leaf expansion but inhibited root growth in tobacco (*Nicotiana tabacum*) (Werner et al., 2001) and the inhibitory effects of exogenous CK on lateral root formation (Laplaze et al., 2007). However, the cytokinin-dependent systemic N-signaling was required for responses to nitrate-rich supply, while another important element identified as part of this systemic N signal in roots under N deficiency was small signaling C-TERMINALLY ENCODED PEPTIDES (CEPs) (Tabata et al., 2014; Ohkubo et al., 2017; Vega et al., 2019). Ryu et al. (2003) found a role for the cytokinin-signaling pathway in growth promotion by GB03. In microarray experiments, the differential expression of the enzyme involved in cytokinin biosynthesis was observed between GB03 exposure and the control (Zhang et al., 2007). Additionally, microarray analyses had demonstrated that CK negatively regulated nitrogen uptake-related genes (Liu et al., 1999; Orsel et al., 2004; Li et al., 2006; Lin et al., 2008). In our work, CK content was decreased by GB03 compared with the water control under TN (**Figure 4B**); on the contrary, root total nitrogen content was increased under the same treatment (**Figure 3B**). Therefore, the reduction in CK content caused by GB03 may have promoted nitrogen uptake in the root, which finally contributed to enhance root and shoot growth (**Figure 5C**). Similarly, it had been reported that CK negatively regulated other nutrient acquisition-related genes in *Arabidopsis*, such as high-affinity phosphate transporter genes (Martin et al., 2000; Sakakibara et al., 2006), consistent with our result that the root total phosphorus content was increased by 10% under TN with GB03 compared to water control (**Figure 3D**). Interestingly, GB03 had no effect on CK content compared with the water control under LN conditions (**Figure 4B**), nor did it affect nitrogen content (**Figure 3B**) or root biomass (**Figures 2E, F**). In this context, they proposed that the role of GB03 promoted tall fescue growth by regulating CK which was nitrogen-dependent (**Figures 5C, D**). We also observed that the effect of auxin on root architecture was greater than that of CK.

In recent years, a connection between other phytohormone (including gibberellic acid (GA), brassinosteroid (BR), and jasmonate) and nitrate responses had been proposed using transcriptome approaches (Canales et al., 2014; Gaudinier et al., 2018; Varala et al., 2018). To date, nitrate and GA had been found to be associated with the modulation of flowering time (Gras et al., 2018), but not with shoot growth and root morphological development. In our study, NO_3_^-^ levels had no effect on GA content in tall fescue without GB03; however, compared with water control, it showed diverse arrays of GA content responses to different NO_3_^-^ concentrations with GB03 (**Figure 4C**). In microarray experiments, two gibberellin-responsive proteins and an enzyme involved in gibberellin biosynthesis were identified as differentially expressed by GB03 in *A. thaliana* (Zhang et al., 2007). Consistent with this result, GA content in tall fescue was increased by exposure to GB03 under TN treatment but decreased under LN treatment (**Figure 4C**). Nevertheless, early in the 19th century, to probe the mechanism underlying PGPR strain GB03 to enhance plant growth, it was tested against a series of *A. thaliana* mutants defective in specific regulatory pathways, and it was observed that enhanced total leaf surface area resulted from exposure to GB03 both for *Arabidopsis thaliana* WTs (Col-0, C-24, and Wassilewskija) and gibberellic acid-insensitive *gai2* mutants (Ryu et al., 2003). Taken together, we initially negated the essential involvement of the gibberellic acid-signaling pathway in the activation of growth promotion and tolerance of nitrogen starvation by GB03 in tall fescue.

Different from IAA, CK, and GA, ABA is generally known as a stress hormone involved in abiotic and biotic stress responses (Kiba et al., 2011). The results presented here showed that ABA content increased by 37.5% under nitrogen deficiency (**Figure 4D**). It was found that the primary root lengths of two *Arabidopsis* ABA-insensitive mutants *abi4-1* and *abi4-2* were both shorter than that of the wild type under 0.1 mM KNO_3_; in contrast, no prominent differences had been observed between mutants and WT among various high KNO_3_ concentrations (1, 10, and 50 mM), indicating the role of ABA in simulating primary root growth under low NO_3_^-^ supply (Signora et al., 2001). Ondzighi-Assoume et al. (2016) revealed a root tip ABA accumulation pattern and demonstrated a mechanism for nitrate-mediated root growth *via* regulation of ABA accumulation in the primary root tip. As shown in our model, the root length of tall fescue was increased by 83.8% under LN, removing the effect of apical accumulation of auxin; the increase in ABA content also contributed it (**Figure 5**). Mining microarray data revealed a cluster of ABA-synthesized and ABA-responsive genes that were downregulated in *A. thaliana* upon exposure to GB03 (Zhang et al., 2008a). Consistent with the transcriptional reduction, ABA content was decreased by exposure to GB03 in our study (**Figure 4D**). This reduction in tissue accumulation of ABA fully explained the results presented here, in that the growth of tall fescue was enhanced by GB03, even under nitrogen deficiency stress (**Figures 1**, **2**). Simultaneously, it explained well why GB03 could effectively alleviate primary root elongation at a low nitrogen level. The large decrease in ABA accumulation greatly inhibited primary root growth in the apical region, despite showing the relatively high accumulation of auxin at the same time (**Figure 5D**). Ristova et al. (2016) combinatorial experiments with auxin, cytokinin, ABA, and nitrate showed that ABA was the main stimulus shaping root system architecture. As a consequence, the inhibitory effect of ABA on root elongation was greater than the promoting effect of IAA under LN with GB03. In addition, the reduced ABA level was also necessary for GB03-enhanced plant photosynthetic activity (Zhang et al., 2008a). Photosynthesis converted light energy into chemical energy in the form of energy-rich sugar molecules; conversely, elevated sugar levels in plant induced storage processes and conferred feedback inhibition of photosynthesis (Rook et al., 2001; Moore et al., 2003; Rolland et al., 2006). Hexokinases were evolutionarily conserved glucose sensors in eukaryotes (Rolland et al., 2006). Studies using *Arabidopsis* mutants defective in hexokinase-dependent sugar signaling indicated that GB03 augmented photosynthesis by repressing hexokinase-dependent, rather than hexokinase-independent, sugar signaling. Glucose signaling largely overlapped with ABA signal transduction, as revealed by the fact that *Arabidopsis* ABA synthesis mutant *aba* and ABA-insensitive mutant *abi* were also, to varying degrees, sugar-sensing mutants (Smeekens, 2000; Rolland et al., 2006). Therefore, the reduction in ABA levels (**Figure 4D**) indirectly explained the improved chlorophyll concentration (**Table 1**), which could serve as one of the parameters for enhancing tall fescue photosynthetic capacity, in GB03-exposed plants (**Figures 5C, D**).

Tall fescue has strong adaptability to temperature and soil conditions, so it is widely used in turf grass planting in China, including Zhejiang, northern regions of Jiangxi, Hunan, Hubei, Jiangsu, Shanghai, Anhui, Shandong, Henan, Hebei, Shanxi, Beijing, Tianjin, and other places (Chen and Zhu, 2006). Nitrogen fertilizer is the most required nutrient for turfgrass, which can effectively improve turfgrass quality and yield. With the steady increase in nitrogen fertilizer application, the problem of low nitrogen fertilizer utilization rate has attracted more and more attention. According to relevant data, China accounts for one-third of the global chemical fertilizer application; the average nitrogen fertilizer utilization rate is only 27%–28%, or even lower, and the loss rate of nitrogen fertilizer application is 45% (Zhang, 2019). Therefore, it is imperative to improve the utilization efficiency of nitrogen fertilizer. Soil microorganisms usually increase nitrogen utilization in plants, especially in legumes. However, the mechanism of their action in grasses is still lacking. Our study showed, for the first time, that the soil bacteria GB03 played a crucial role in mediating the nitrogen-dependent regulation of tall fescue growth and development by facilitating photosynthesis, nutrient accumulation, and phytohormone homeostasis at the physiological level, although the underlying cellular and molecular mechanisms of action remain unclear. Therefore, the cell size and morphology as well as tall fescue genome from various tissues under different treatments will be needed, which can more systematically illuminate the mechanism of growth-promoting and stress resistance by soil bacteria.

## Data availability statement

The raw data supporting the conclusions of this article will be made available by the authors, without undue reservation.

## Author contributions

QW designed the project, performed experiments, collected data, analyzed results, and wrote up the study. E-LO performed experiments, collected data, and analyzed results. YC performed experiments and collected data. Z-YW performed experiments. Z-WW collected data. P-CW analyzed the results and revised this paper. X-WF designed the project and analyzed the results. J-LZ designed the project, analyzed results, and wrote up the study. All authors contributed to the article and approved the submitted version.

## Funding

This research was supported by the National Nature Science Foundation of China (31802128), the Qian Academy of Agricultural Sciences post-subsidy of the National Nature Science Foundation (qiannongkeyuanguojihoubuzhu[2021]25), the Guizhou Province Science and Technology Planning Project (qiankehejichu[2020]1Z027), the Guizhou Province Hundred-level talent Project (qiankehepingtairencai-GCC[2022]022-1), and the Qian Academy of Agricultural Sciences post-subsidy of the National Nature Science Foundation (qiannongkeyuanguojihoubuzhu[2021]13).

## Conflict of interest

The authors declare that the research was conducted in the absence of any commercial or financial relationships that could be construed as a potential conflict of interest.

## Publisher’s note

All claims expressed in this article are solely those of the authors and do not necessarily represent those of their affiliated organizations, or those of the publisher, the editors and the reviewers. Any product that may be evaluated in this article, or claim that may be made by its manufacturer, is not guaranteed or endorsed by the publisher.

## Supplementary material

Supplementary Figure 1 Effects of DH5α and GB03 on tall fescue phenotype. **(A)** Individual plant and **(B)** one pot of plant (containing five seedlings) were photographed from different parallel treatments, including the total nitrogen level (TN-3.75 mM NO_3_^-^) and the low nitrogen level (LN-0.25 mM NO_3_^-^) with DH5α or GB03, respectively, and the water treatment as control.

Supplementary Table 1 Effects of DH5α and GB03 on tall fescue growth under various concentrations of NO_3_^-^. The treatments were: total nitrogen (TN), total nitrogen with *E. coli* DH5α (TN + DH5α), total nitrogen with soil bacteria GB03 (TN + GB03), low nitrogen stress (LN), low nitrogen stress with *E. coli* DH5α (LN + DH5α) and low nitrogen stress with soil bacteria GB03 (LN + GB03). Values are means ± SE (*n* = 8). The different letters indicate significant differences at *p* < 0.05 (Duncan’s test).
