# Supplementary figures and images for: Bacillus amyloliquefaciens GB03 augmented tall fescue growth by regulating phytohormone and nutrient homeostasis under nitrogen deficiency
Source: Front Plant Sci. 2022 Oct 6;13:979883. doi: 10.3389/fpls.2022.979883 (PMC9582836; doi:10.3389/fpls.2022.979883)

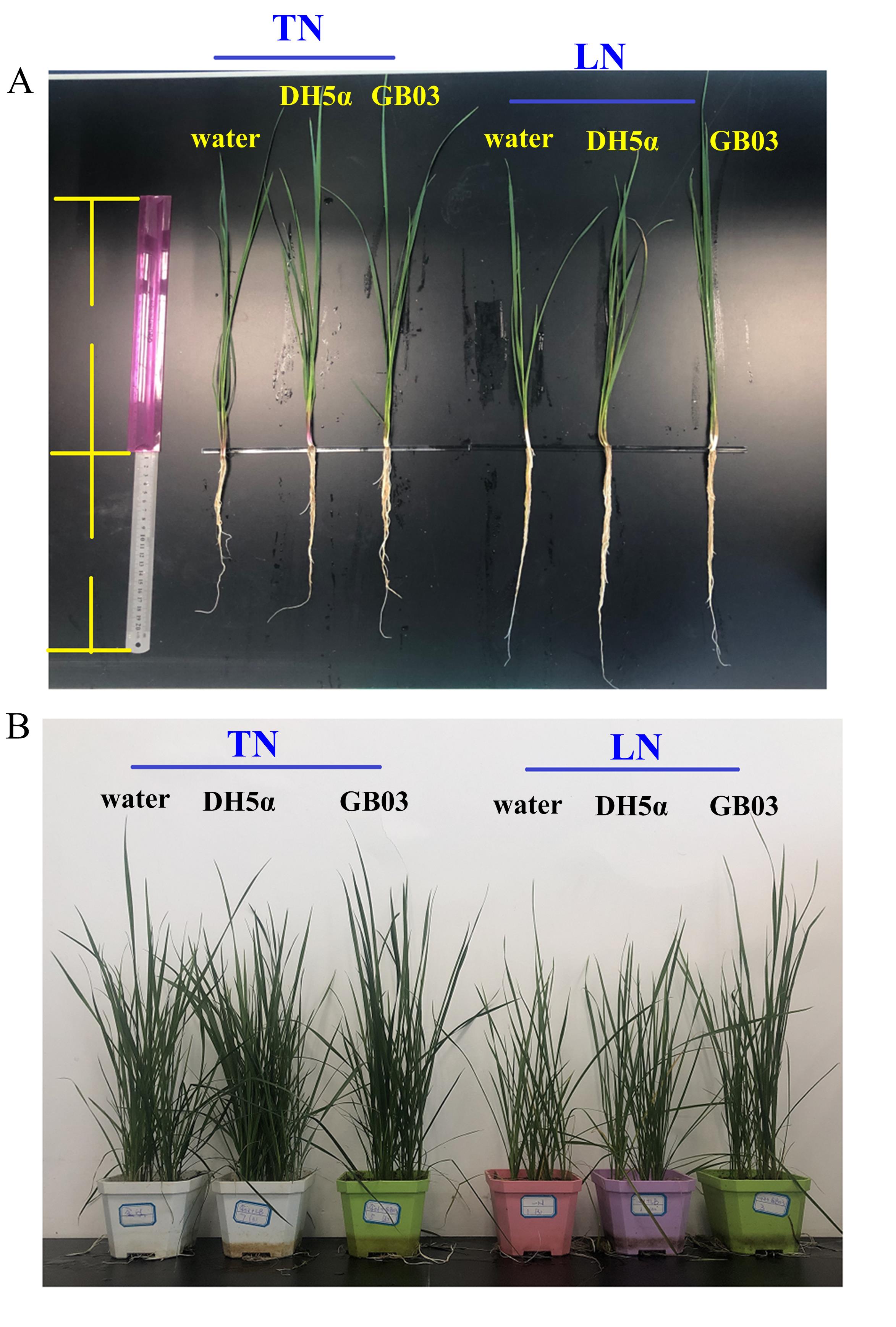

Supplement: Supplementary Figure 1 — Effects of DH5α and GB03 on tall fescue phenotype. (A) Individual plant and (B) one pot of plant (containing five seedlings) were photographed from different parallel treatments, including the total nitrogen level (TN-3.75 mM NO3 -) and the low nitrogen level (LN-0.25 mM NO3 -) with DH5α or GB03, respectively, and the water treatment as control. [file Image_1.jpeg]
