# Supplementary material for: Bacillus amyloliquefaciens GB03 augmented tall fescue growth by regulating phytohormone and nutrient homeostasis under nitrogen deficiency
Source: Front Plant Sci. 2022 Oct 6;13:979883. doi: 10.3389/fpls.2022.979883 (PMC9582836; doi:10.3389/fpls.2022.979883)
Supplement: Supplementary Table 1 — Effects of DH5α and GB03 on tall fescue growth under various concentrations of NO3 -. The treatments were: total nitrogen (TN), total nitrogen with E. coli DH5α (TN + DH5α), total nitrogen with soil bacteria GB03 (TN + GB03), low nitrogen stress (LN), low nitrogen stress with E. coli DH5α (LN + DH5α) and low nitrogen stress with soil bacteria GB03 (LN + GB03). Values are means ± SE (n = 8). The different letters indicate significant differences at p < 0.05 (Duncan’s test). [file Table_1.docx]

**SUPPLEMENTARY TABLE. 1 Effects of DH5α and GB03 on tall fescue growth under various concentrations of NO_3_**^-^. The treatments were: total nitrogen (TN), total nitrogen with *E. coli* DH5α (TN + DH5α), total nitrogen with soil bacteria GB03 (TN + GB03), low nitrogen stress (LN), low nitrogen stress with *E. coli* DH5α (LN + DH5α) and low nitrogen stress with soil bacteria GB03 (LN + GB03). Values are means ± SE (*n* = 8). The different letters indicate significant differences at *p* < 0.05 (Duncan’s test).

| Treatments | Plant height  (cm) | Root length (cm) | SFW  (g/plant) | SDW (g/plant) | RFW  (g/plant) | RDW  (g/plant) |
| --- | --- | --- | --- | --- | --- | --- |
| TN | 32.89 ± 0.84 b | 12.11 ± 0.32 d | 1.77 ± 0.07 b | 0.22 ± 0.006 b | 0.39 ± 0.04 c | 0.03 ± 0.003 c |
| TN + DH5α | 33.00 ± 0.85 b | 12.67 ± 0.46 cd | 1.81 ± 0.05 b | 0.22 ± 0.004 b | 0.38 ± 0.03 c | 0.03 ± 0.004 c |
| TN + GB03 | 37.50 ± 0.70 a | 13.70 ± 0.53 c | 2.44 ± 0.09 a | 0.31 ± 0.013 a | 0.67 ± 0.06 a | 0.06 ± 0.006 a |
| LN | 25.88 ± 0.95 c | 22.25 ± 1.29 a | 0.81 ± 0.05 d | 0.11 ± 0.007 d | 0.42 ± 0.04 bc | 0.04 ± 0.003 bc |
| LN + DH5α | 26.88 ± 1.16 c | 20.86 ± 0.70 ab | 0.86 ± 0.04 d | 0.11 ± 0.007 d | 0.50 ± 0.04 bc | 0.04 ± 0.003 bc |
| LN + GB03 | 33.56 ± 1.05 b | 14.50 ± 0.33 c | 1.07 ± 0.05 c | 0.14 ± 0.005 c | 0.53 ± 0.04 b | 0.05 ± 0.003 ab |
